# Supplementary material for: Adherens junctions limit septate junction length in Drosophila midgut enterocytes but are not required for polarity
Source: J Cell Sci. 2025 Jul 11;138(13):jcs263644. doi: 10.1242/jcs.263644 (PMC12276802; doi:10.1242/jcs.263644)
Supplement: Supplementary information [file joces-138-263644-s1.pdf]

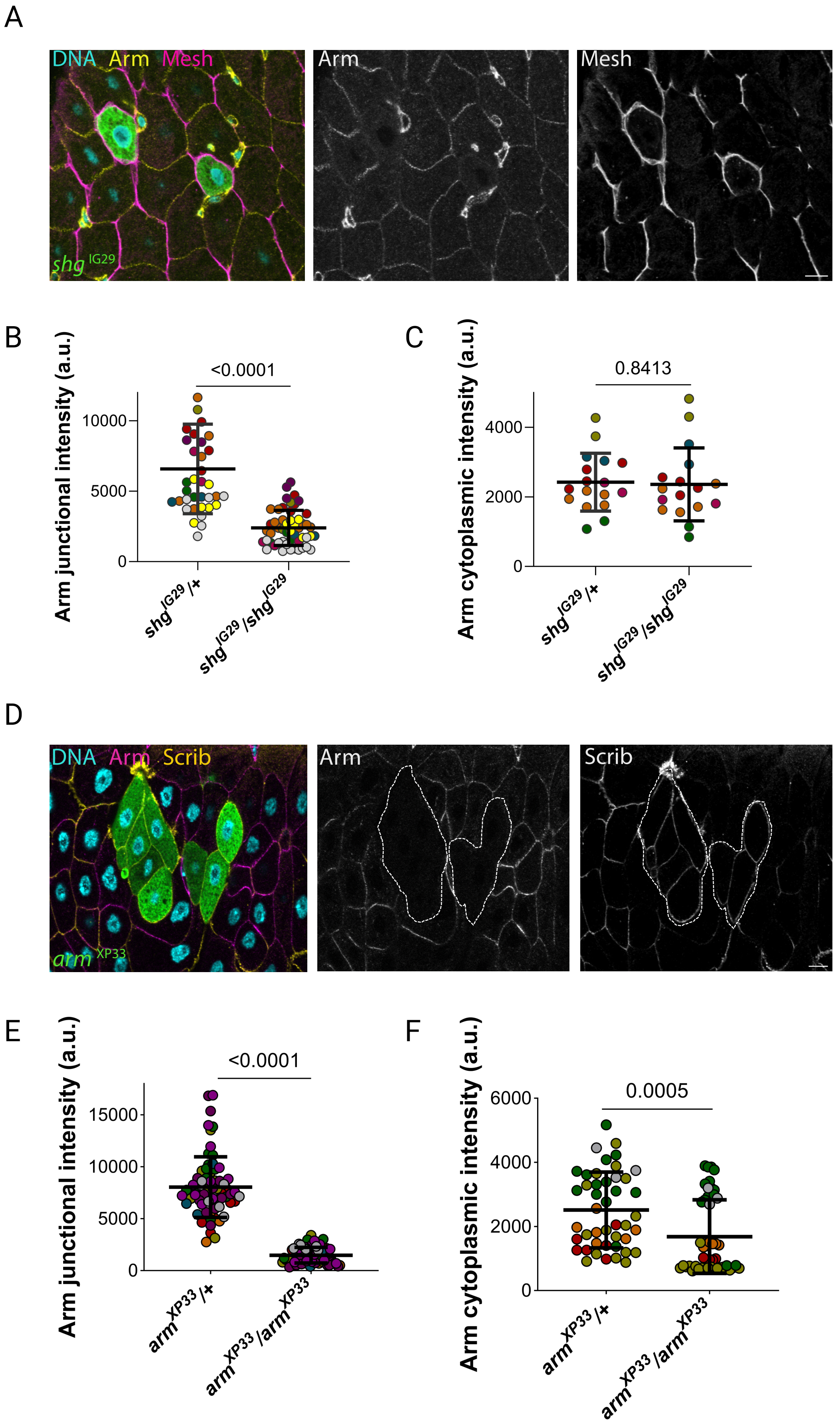

Journal of Cell Science • Supplementary information

Fig. S1. β-catenin is degraded in *shotgun* mutants.

A. A horizontal confocal section near the basal side of a midgut containing a *shg*^{IG29} homozygous clone (GFP; green), stained for DNA (DAPI; blue), Armadillo (magenta) and Mesh (yellow). **B.** A superplot of Armadillo intensity at cell-cell junctions in *shg*^{IG29}/+ (mean=6577±3183, 38 cell-cell junctions, 9 midguts) and *shg*^{IG29}/*shg*^{IG29} (mean=2397±1250, 53 cell-cell junctions, 9 midguts) enterocytes; **C.** A superplot showing Armadillo cytoplasmic intensity in *shg*^{IG29}/+ (mean=2425±832, 17 cells, 6 midguts) and *shg*^{IG29}/*shg*^{IG29} (mean=2360±1047, 17 cells, 6 midguts) enterocytes; **D.** A horizontal confocal section near the basal side of a midgut containing an *arm*^{XP33} homozygous clone (GFP; green), DNA (DAPI; blue), Armadillo (magenta) and Scribble (yellow). **E.** A superplot showing Armadillo intensity at cell-cell junctions between *arm*^{XP33}/+ enterocytes (mean=8037±2920, 67 cell-cell junctions, 7 midguts) and between *arm*^{XP33}/*arm*^{XP33} enterocytes (mean=1487±752, 73 cell-cell junctions, 7 midguts); **F.** A superplot showing Armadillo cytoplasmic intensity in *arm*^{XP33}/+ (mean=2512±1183, 44 cells, 4 midguts) and *arm*^{XP33}/*arm*^{XP33} (mean=1684±1144, 49 cells, 4 midguts) enterocytes. The scale bars in A and D = 10 µm.

Journal of Cell Science • Supplementary information

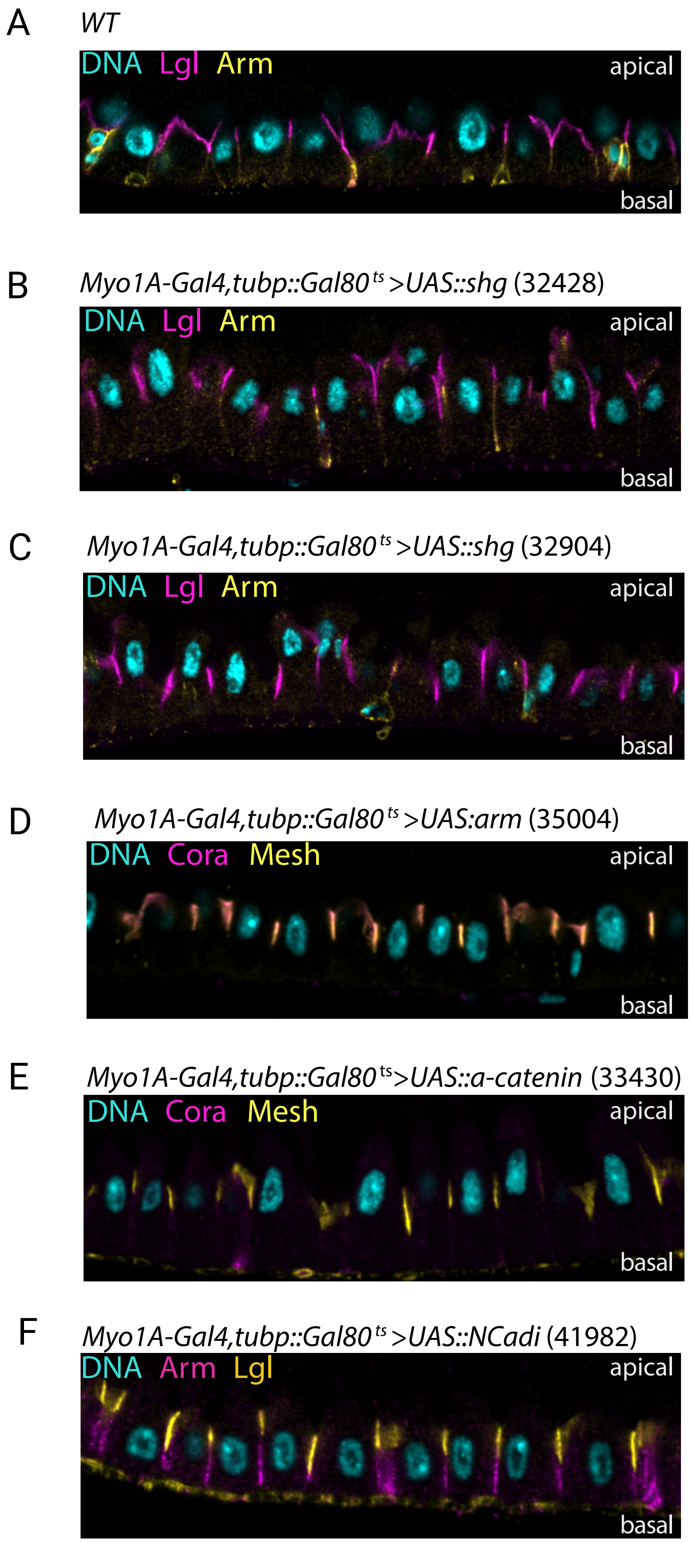

Fig. S2. Adherens junctions are not required for the maintenance of apical-basal polarity in the midgut.

A. An apical-basal section through a wild-type midgut stained for DNA (DAPI; blue), Lgl (magenta) and Armadillo (yellow). **B and C.** Apical-basal sections through midguts expressing different *shotgun* RNAi lines under the control of Myo1A-Gal4, stained for DNA

Journal of Cell Science • Supplementary information

(DAPI; blue), Lgl (magenta) and Armadillo (yellow). **D.** An apical-basal section through a midgut expressing *armadillo* RNAi and stained for DNA (DAPI; blue), Coracle (magenta) and Mesh (yellow). **E.** An apical-basal section through a midgut expressing *α-catenin* RNAi stained for DNA (DAPI; blue), Coracle (magenta) and Mesh (yellow); **F.** An apical-basal section through a midgut expressing *N-cadherin 1* RNAi stained for DNA (DAPI; blue), Armadillo (magenta) and Lgl (yellow).

Journal of Cell Science • Supplementary information

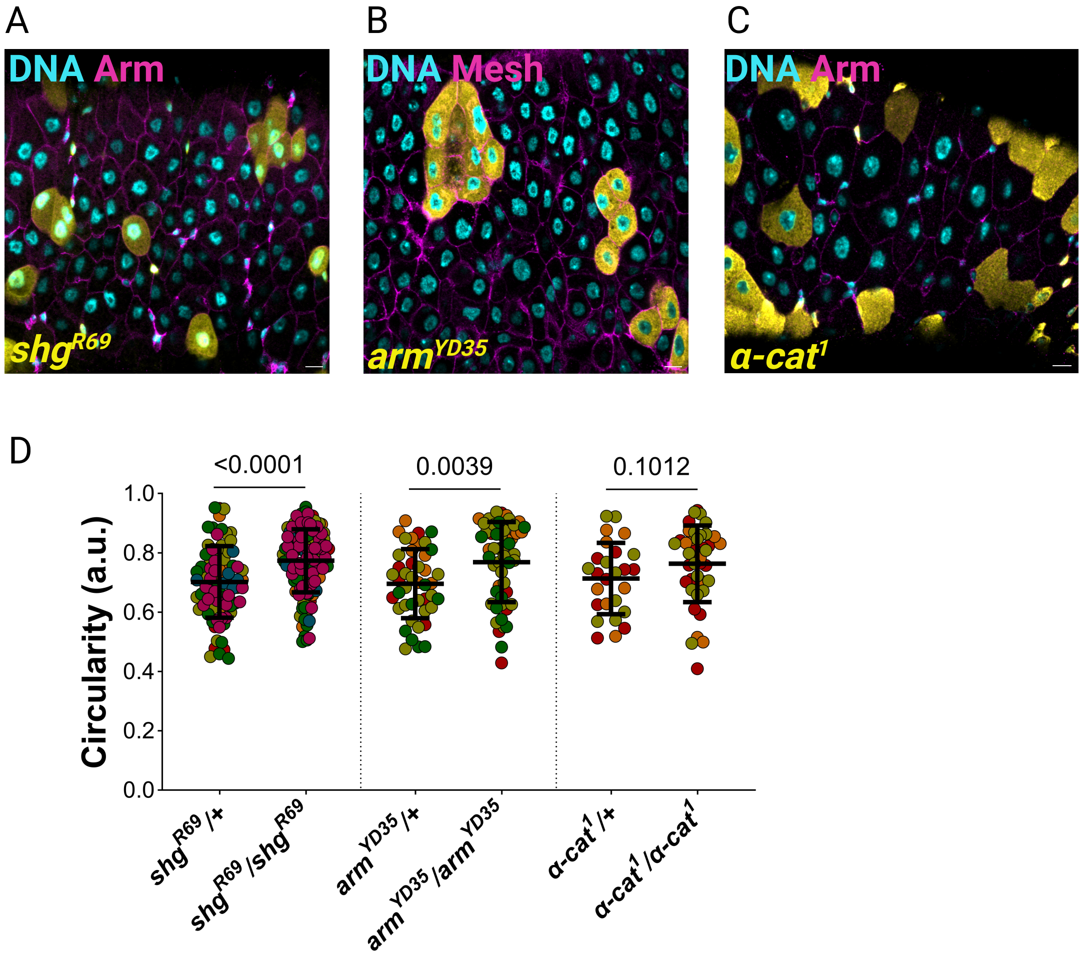

Fig. S3. E-cadherin and β-catenin regulate enterocyte shape independently of α-catenin.

A. Horizontal sections though midguts containing *shg*^{R69} homozygous clones marked by GFP expression (yellow) stained for DNA (DAPI, blue) and Armadillo (magenta). **B.** *arm*^{YD35} homozygous clones (yellow) stained for DNA (DAPI, blue) and Mesh (magenta). **C.** *α-catenin*^{1} homozygous clones (yellow) stained for DNA (DAPI, blue) and Armadillo (magenta). Scale bar=10 µm. **D.** A Superplot showing the circularity at the basal side of *shg*^{R69}/+ (82 cells, 5 midguts), *shg*^{R69}/*shg*^{R69} (129 cells, 5 midguts), *arm*^{YD35}/+ (44 cells, 4 midguts), *arm*^{YD35}/*arm*^{YD35} (52 cells, 4 midguts), *α-cat*^{1}/+ (26 cells, 3 midguts) and *α-cat*^{1}/*α-cat*^{1} (40 cells, 3 midguts) enterocytes. The scale bars from A to C= 10 µm.

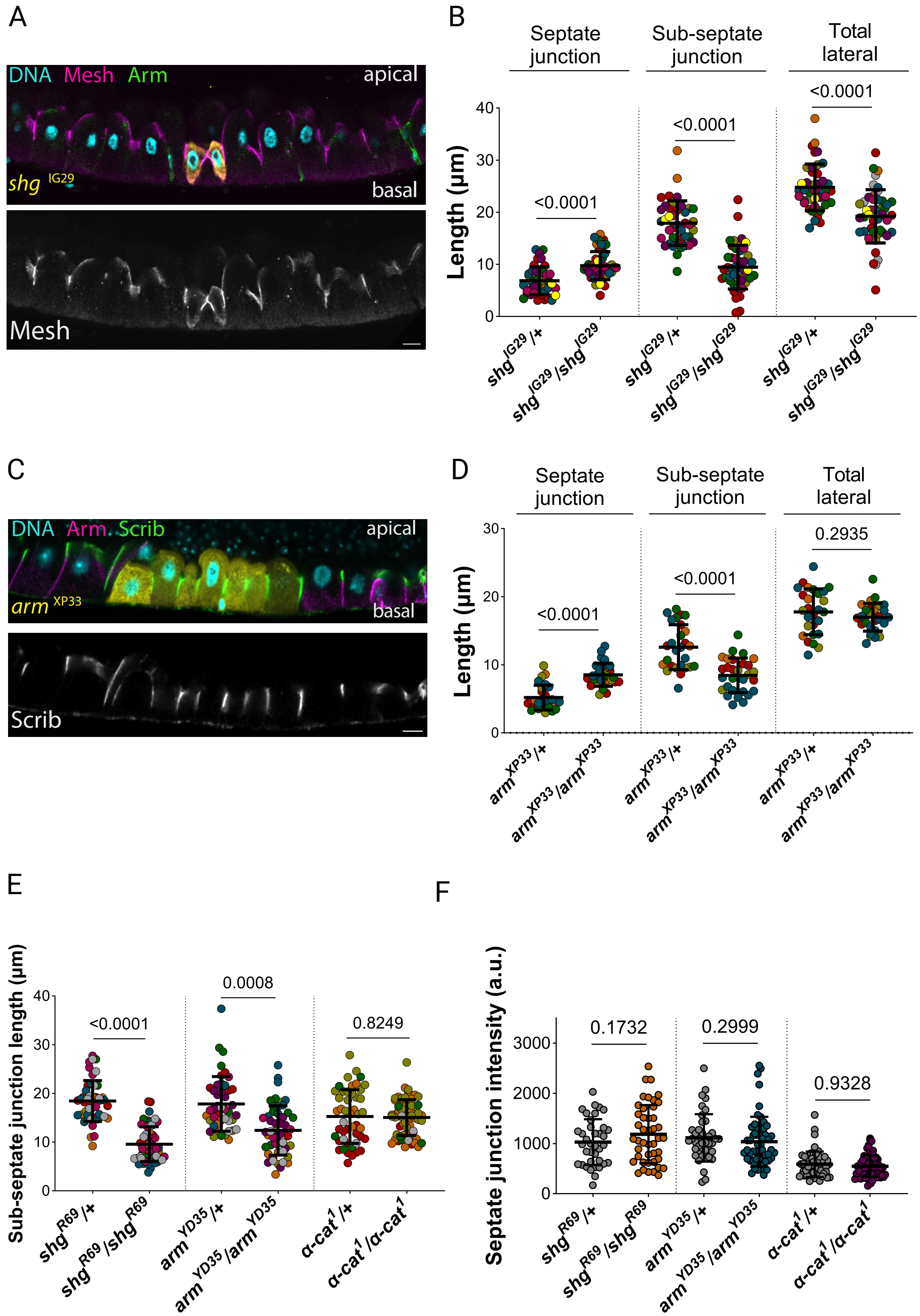
A
DNA Mesh Arm
apical
shg IG29
basal
Mesh
B
Septate junction
Sub-septate junction
Total lateral
Length (µm)
<0.0001
<0.0001
<0.0001
shg IG29 /+
shg IG29 /shg IG29
C
DNA Arm Scrib
apical
arm XP33
basal
Scrib
D
Septate junction
Sub-septate junction
Total lateral
Length (µm)
<0.0001
<0.0001
0.2935
arm XP33 /+
arm XP33 /arm XP33
E
Sub-septate junction length (µm)
<0.0001
0.0008
0.8249
shg R69 /+
shg R69 /shg R69
arm YD35 /+
arm YD35 /arm YD35
α-cat 1 /+
α-cat 1 /α-cat 1
F
Septate junction intensity (a.u.)
0.1732
0.2999
0.9328

Fig. S4. E-cadherin and β-catenin regulate Septate junction length.

A. An apical-basal section of a midgut containing homozygous cells mutant for a second *shotgun* allele, shg^{IG29} (yellow) stained for DNA (DAPI, blue) and Mesh (magenta). **B.** Measurements of the lengths of the septate junctions (shg^{IG29}/+: 6.86±2.67µm, 43 junctions; shg^{IG29}/shg^{IG29}: 9.75±2.70µm, 46 junctions, 8 midguts), the sub-septate junction domain (shg^{IG29}/+: 17.91±4.29µm, 43 junctions; shg^{IG29}/shg^{IG29}: 9.47±4.20µm, 46 junctions, 8 midguts) and the total lateral length (shg^{IG29}/+: 24.77±4.47µm, 43 junctions; shg^{IG29}/shg^{IG29}: 19.22±5.11µm, 46 junctions, 8 midguts) in shg^{IG29} heterozygous and homozygous enterocytes. **C.** An apical-basal section of a midgut containing a homozygous mutant clone for a second *arm* allele arm^{XP33} (yellow) stained for DNA (DAPI; blue), Armadillo (magenta) and Scribble (yellow). **D.** A superplot showing the lengths of the septate junctions (arm^{XP33}/+: 5.19±1.82µm, 27 junctions; arm^{XP33}/arm^{XP33}: 8.54±1.70µm, 29 junctions, 5 midguts), sub-septate junction domain (arm^{XP33}/+: 12.59±3.30µm, 27 junctions; arm^{XP33}/arm^{XP33}: 8.45±2.53µm, 29 junctions, 5 midguts) and the total lateral length (arm^{XP33}/+: 17.78±3.37µm, 27 junctions; arm^{XP33}/arm^{XP33}: 16.98±2.03µm, 29 junctions, 5 midguts) in arm^{XP33}/+ and ; arm^{XP33}/arm^{XP33} enterocytes. **E.** A superplot showing the length of the sub-septate junction domain in shg^{R69}/+ (6 midguts, 39 junctions), shg^{R69}/shg^{R69} (6 midguts, 46 junctions), arm^{YD35}/+ (7 midguts, 44 junctions), arm^{YD35}/arm^{YD35} (7 midguts, 50 junctions), $\alpha\text{-}cat^{1}$/+ (4 midguts, 47 junctions) and $\alpha\text{-}cat^{1}/\alpha\text{-}cat^{1}$ (4 midguts, 61 junctions) enterocytes. **F.** A superplot showing the intensity of septate junction proteins per unit length in shg^{R69}/+ (mean=1027±458.8, 6 midguts, 39 junctions), shg^{R69}/shg^{R69} (mean=1186±579.4, 6 midguts, 46 junctions), arm^{YD35}/+ (mean=1114±472.9, 7 midguts, 44 junctions), arm^{YD35}/arm^{YD35} (mean=1036±496.7, 7 midguts, 50 junctions), $\alpha\text{-}cat^{1}$/+ (mean=587±254.4, 6 midguts, 54 junctions) and $\alpha\text{-}cat^{1}/\alpha\text{-}cat^{1}$ (mean=548.5±213.4, 6 midguts, 74 junctions) enterocytes. Scale bars A and C= 10 µm.

Journal of Cell Science • Supplementary information

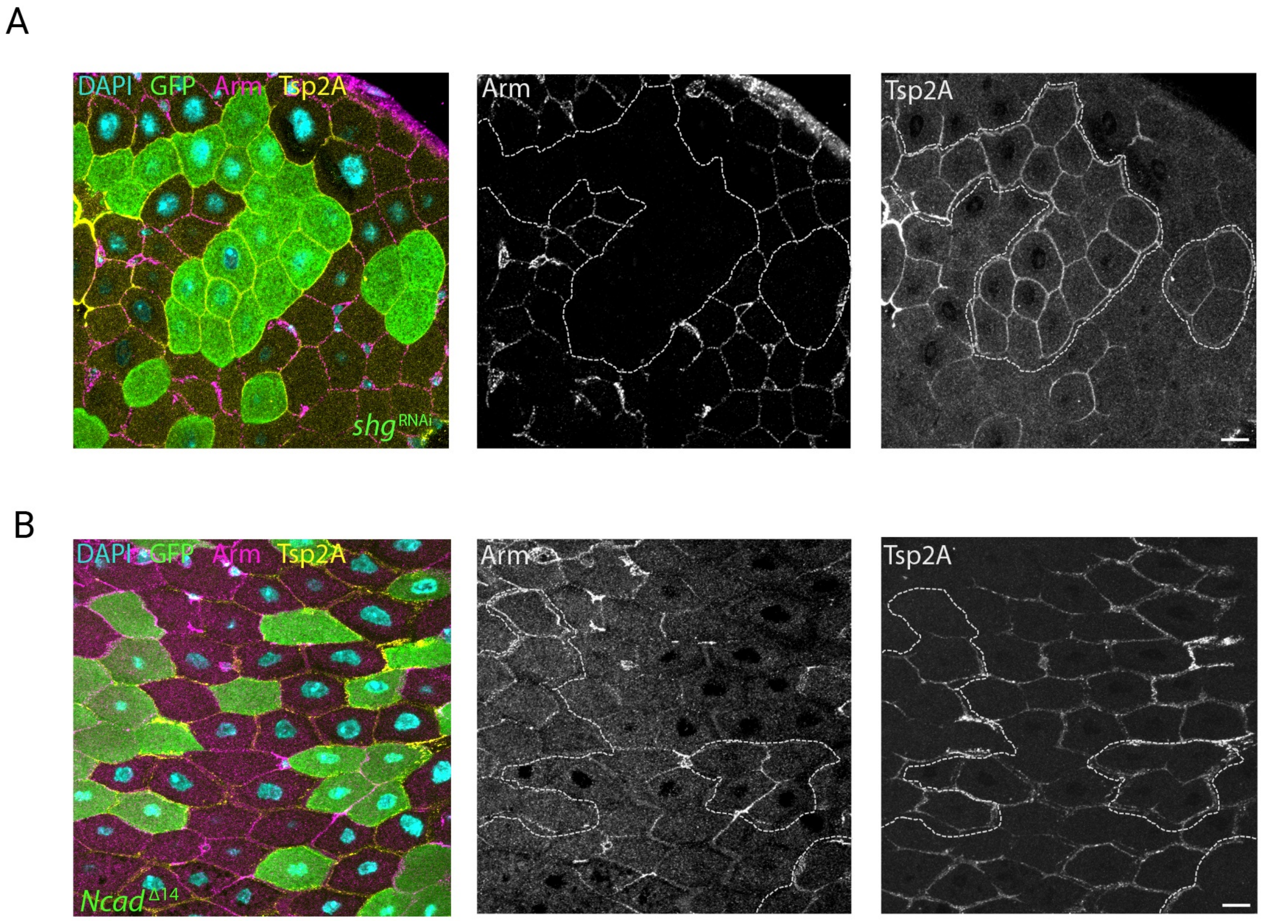

Fig. S5. All three classical cadherins are dispensable for apical-basal polarity of the midgut.

A. A horizontal section through the basal region of a midgut containing MARCM clones expressing *shg*^{RNAi} (GFP; green) stained for DNA (DAPI, blue), Armadillo (magenta) and Tsp2A (yellow); clones are outlined with white dashed lines; Scale bar= 10µm. **B.** A horizontal section through the basal region of a midgut containing *Ncad*$^{\Delta 14}$ homozygous clones (GFP; green) stained for DNA (DAPI, blue), Armadillo (magenta) and Tsp2A (yellow); clones are outlined with a white dashed line; Scale bar= 10µm
